# Associations among serum trimethylamine-N-oxide (TMAO) levels, kidney function and infarcted coronary artery number in patients undergoing cardiovascular surgery: a cross-sectional study

**DOI:** 10.1007/s10157-015-1207-y

**Published:** 2015-12-16

**Authors:** Aki Mafune, Takeo Iwamoto, Yusuke Tsutsumi, Akio Nakashima, Izumi Yamamoto, Keitaro Yokoyama, Takashi Yokoo, Mitsuyoshi Urashima

**Affiliations:** 1 Division of Molecular Epidemiology, Jikei University School of Medicine, Tokyo, Japan; 2 Core Research Facilities, Jikei University School of Medicine, Tokyo, Japan; 3 Division of Nephrology and Hypertension, Department of Internal Medicine, Jikei University School of Medicine, Tokyo, Japan; 4 Department of Anesthesiology, Intensive Care Unit, Saitama Medical Center, Jichi Medical University, Saitama, Japan; 5 Department of Molecular Epidemiology, Jikei University School of Medicine, 3-25-8 Nishi-shimbashi, Minato-ku, Tokyo, 105-8461 Japan

**Keywords:** Glomerular filtration rate (GFR), Trimethylamine-N-oxide (TMAO), Cardiovascular disease (CVD), Coronary artery disease (CAD)

## Abstract

**Background:**

Trimethylamine-N-oxide (TMAO) is a metabolite of phosphatidylcholine generated by gut microbiota and liver enzymes, and has recently been recognized as contributing to atherosclerosis. Elevated serum TMAO levels have been shown to increase the risk of cardiovascular disease (sudden death, myocardial infarction, or stroke) in patients undergoing elective coronary angiography. We aimed to clarify whether TMAO levels are associated with the number of infarcted coronary arteries as a measure of the severity of atherosclerosis, with adjustment using a priori-defined covariates such as kidney function.

**Methods:**

By conducting a cross-sectional study of 227 patients who underwent cardiovascular surgery for coronary artery disease, valvular heart disease, or aortic disease, the association between serum TMAO levels as measured by HPLC-APCI-MS/MS and the number of infarcted coronary arteries was evaluated using ordered logistic regression models with adjustment of 10 covariates, including chronic kidney disease (CKD) stage. Unadjusted and adjusted odds ratios (ORs) and 95 % confidence intervals (95 % CIs) were determined.

**Results:**

Significantly higher TMAO levels were observed in advanced-stage CKD (*p* ≤ 0.001). In fully adjusted models with the 10 covariates, a significantly increased number of infarcted coronary arteries was identified in the highest quartile and quintile of TMAO compared to the lowest quartile (OR 11.9; 95 % CI 3.88–36.7, *p* ≤ 0.001) and quintile (OR 14.1; 95 % CI 3.88–51.2; *p* ≤ 0.001), respectively, independent of dyslipidemia.

**Conclusions:**

Higher serum TMAO levels may be associated with advanced CKD stages and with an increased number of infarcted coronary arteries in patients who undergo cardiovascular surgery.

## Introduction

Eggs, milk, liver, red meat and fish represent rich sources of dietary phosphatidylcholine. Phosphatidylcholine is metabolized by gut microbiota and hepatic flavin-containing monooxygenase 3 (FMO3) to trimethylamine-N-oxide (TMAO). An animal study has found that TMAO enhances cholesterol accumulation in macrophages and in foam cells in artery walls, which contributes to atherosclerosis that leads to cardiovascular disease [1]. A cohort study has already shown that higher serum TMAO levels were associated with an increased risk of incident major adverse cardiovascular events (death, myocardial infarction, or stroke) during 3 years of follow-up in 4007 patients undergoing elective coronary angiography [2], which was further enhanced in combination with high levels of L-carnitine in another cohort study [3]. In contrast, we aimed to clarify whether TMAO levels were associated not with cardiovascular disease, but with the number of infarcted coronary arteries as a measure of the severity of atherosclerosis, adjusting with a priori-defined covariates including chronic kidney disease (CKD) stage in patients who underwent cardiovascular surgery for coronary artery disease (CAD), valvular heart disease (VHD), or aortic disease (AoD), with a post hoc cross-sectional analysis of our previous study [4].

## Materials and methods

### Study design

This cross-sectional study was performed at the intensive care unit of Jichi Medical University Saitama Medical Center. Data were monitored at the Division of Molecular Epidemiology, Jikei University School of Medicine. Planned analyses proceeded in full compliance with the principles of the Declaration of Helsinki. The study protocol was reviewed and approved by the ethics committees of Jichi Medical University School of Medicine (approval #09-23) and Jikei University School of Medicine (approval #21-184 6062).

### Study population

**Fig. 1:**
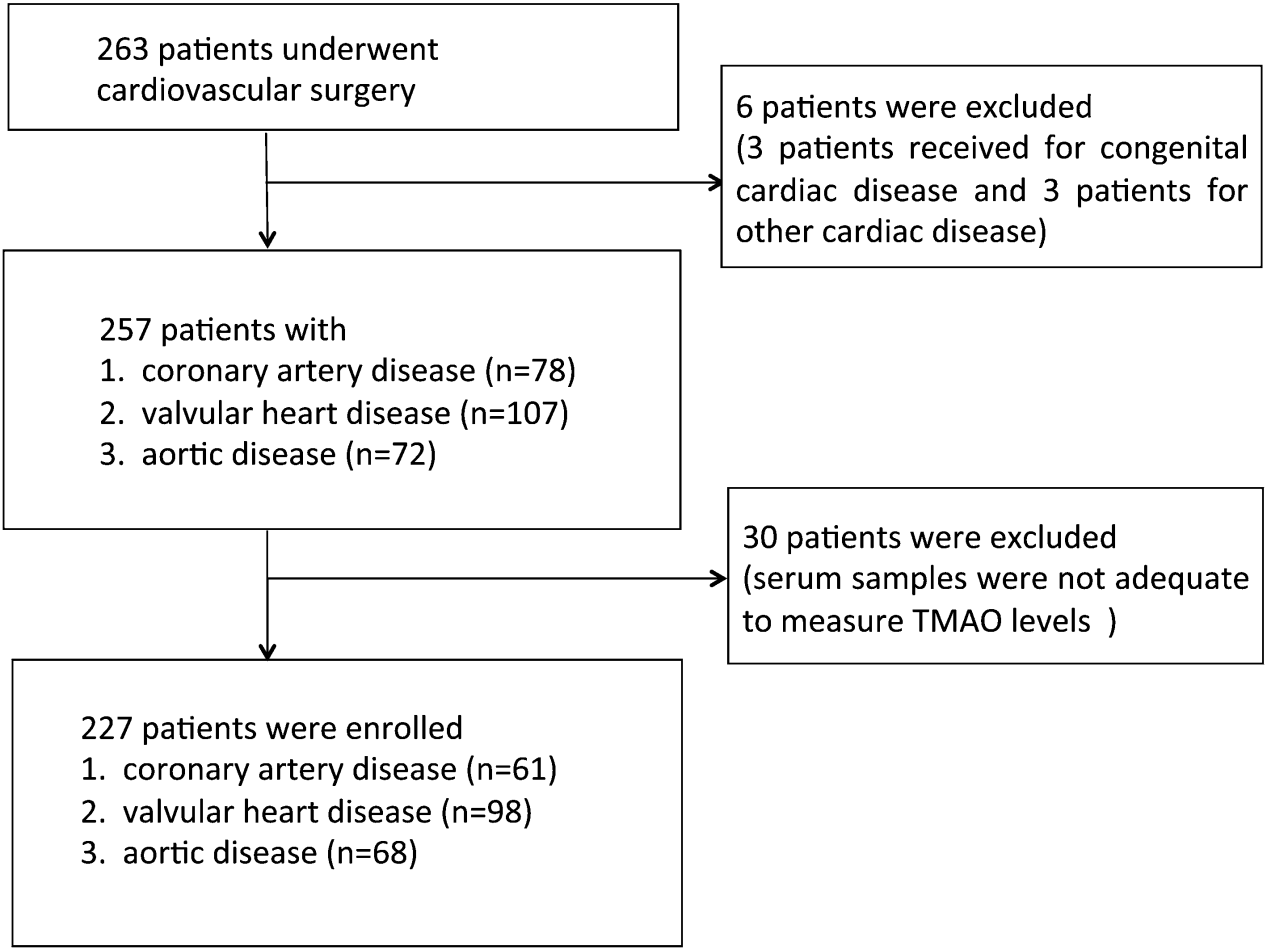
Patient flow chart

The patient flow chart is shown as Fig. 1. A total of 263 consecutive patients were scheduled to undergo major cardiovascular surgery at Jichi Medical University Saitama Medical Center during the study period from January 28, 2010 to October 29, 2010. Eligible patients had operations for CAD, including unstable angina and acute myocardial infarction, VHD including aortic stenosis, aortic regurgitation, mitral stenosis, mitral regurgitation and tricuspid regurgitation, or AoD including thoracic/abdominal aortic aneurysm and aortic dissection, diagnosed by cardiac surgeons according to the American College of Cardiology/American Heart Association guideline. On the other hand, six patients with congenital and other non-atherosclerotic cardiac diseases were excluded. The quantity of serum samples from 30 patients was too small to measure TMAO levels; thus, data from the remaining 227 patients were analyzed. The day before surgery, the anesthesiologist in charge explained all procedures associated with the study and its purpose to the patients, who provided their written, informed consent to participate.

### Clinical evaluation

Clinical information such as baseline characteristics, comorbidity factors, various clinical scores, and medical history was extracted from medical records and pre-anesthesia interview forms. Age and sex were checked in the clinical chart. Body mass index (BMI) (kg/m^2^) was calculated as weight divided by the square of height. Laboratory data for serum creatinine (Cr) and low-density lipoprotein-cholesterol (LDL-C) were collected in the outpatient clinic before surgery in the fasting state. Estimated glomerular filtration rates (eGFR) (mL/min/1.73 m^2^) were calculated separately for men (eGFR = 194 × Cr^−1.094^ × age^−0.287^) and women (eGFR = 194 × Cr^−1.094^ × age^−0.287^ × 0.739) [5]. CKD stages were defined based on eGFR levels as follows: Stage 1 CKD, eGFR ≥90 ml/min/1.73 m^2^; Stage 2 CKD, eGFR ≥60 to <90 ml/min/1.73 m^2^; Stage 3 CKD, eGFR ≥30 to <60 ml/min/1.73 m^2^; Stage 4 CKD, eGFR ≥15 to <30 ml/min/1.73 m^2^; Stage 5 CKD, eGFR <15 ml/min/1.73 m^2^; and Stage 5D, patients treated with hemodialysis. Pre-operative use of β-blockers, angiotensin-converting enzyme inhibitors (ACEIs), angiotensin II receptor blockers (ARBs), statins, and insulin was checked. The following definitions were used: diabetes, fasting plasma glucose ≥126 mg/dL, HbA1c ≥6.5 % based on the Japanese Diabetes Society, or medication with insulin or hypoglycemic agents; hypertension, systolic blood pressure ≥140 mmHg, diastolic blood pressure ≥90 mmHg, or medication with antihypertensive agents [6]; dyslipidemia, fasting LDL-C ≥140 mg/dL, triglycerides ≥220 mg/dL, or medication with lipid-altering agents [7]; and hemodialysis, maintenance hemodialysis before cardiovascular surgery. The pre-operative clinical and physical status of each patient was evaluated using the New York Heart Association (NYHA) guidelines [8].

### Outcome measure

The number of infarcted coronary arteries was determined using coronary angiography by cardiac surgeons before the cardiovascular surgery, which was used as the outcome measure in this study.

### Exposure measure of TMAO

Serum TMAO levels were measured in a post hoc analysis of our previous study [4]. Serum samples for TMAO measurements were taken in the fasting state, and were collected in the operating room before the induction of anesthesia to avoid the dilution effect of intravenous fluid infusion and stored at −80 °C. TMAO levels were quantified using stable-isotope dilution high-performance liquid chromatography-atmospheric pressure chemical ionization tandem mass-spectrometry (HPLC-APCI-MS/MS) in positive multiple-reaction monitoring (MRM) mode using deuterated internal standards on a QTRAP triple-quadruple mass spectrometer (AB SCIEX, Framingham, MA) and trimethylamine-d9 N-oxide (d9-TMAO) (CAS number #1161070-49-0, Sigma-Aldrich Corp., St. Louis, MO) as the internal standard.

To 100 μL of human serum samples at room temperature were added 429 μL of isopropanol, 286 μL of acetonitrile, and 286 μL of Milli-Q water (Millipore, Bedford, MA), followed by the internal standard d9-TMAO. Mixtures were separated by centrifugation for 10 min at 13,360×*g* (Microcentrifuge 5415R, Eppendorf Inc., Enfield, CT), and supernatant samples (500 μL) were transferred to 1.5-mL Eppendorf tubes. The supernatants were evaporated to dryness using a centrifugal concentrator CC-105 (Tomy Tech USA, Fremont, CA) for 10 h, resuspended in 40 μL of ultrapure water (Wako Pure Chemical Industries, Tokyo, Japan), and vortex-mixed for 10 s. Supernatant (10 μL) was directly injected into the HPLC-APCI-MS/MS system.

Serum TMAO was quantified by HPLC using an Agilent 1100 series (Life Technologies, Santa Clara,CA) and column (CAPCELL CORE ADME S2.7, 2.1 × 75 mm, Shiseido, Tokyo, Japan) in positive mode. Separation proceeded using a linear gradient of 1 % formic acid (A) and 100 % methanol (B) as follows: 1 % formic acid for 0.5 min, then 100:0 (A:B) to 40:60 (A:B) for 6 min and 40:60 (A:B) to 100:0 (A:B) for 6.1 min at a flow rate of 0.3 mL/min at 20 °C. The HPLC instrument was coupled to a QTRAP triple-quadruple mass spectrometer (Life Technologies) with atmospheric pressure chemical ionization. The precursor-production ion pairs used in MRM mode were: *m*/*z* 76 → 59 for TMAO and *m*/*z* 85 → 68 for the internal standard. Other mass spectrometer parameters included ion spray voltage 5000 V and vaporizer temperature 400 °C. Data acquisition was controlled using Analyst 1.6.1 software (AB SCIEX). The calibration standard was 150 μL of TMAO dissolved in ultrapure water. The concentrations of the calibration standards were 2–50 μM TMAO. A calibration curve was constructed by plotting the peak area ratio of TMAO to the internal standard against its concentration. Accuracy is expressed as a ratio (%) of the nominal concentration, and precision is expressed as relative standard deviation. Fresh calibration standards were prepared every week, and all remained stable during the analysis.

### Statistical analysis

The covariates were defined a priori as: (1) age; (2) sex; (3) smoking status divided into three categories (never, previous, and current smoker); (4) BMI; (5) CKD stage; (6) diabetes; (7) HbA_1c_; (8) insulin use; (9) hypertension; (10) ACEI or ARB use; (11) dyslipidemia; (12) LDL-C; (13) statin use; (14) congestive heart failure; (15) NYHA; (16) β-blocker use; and (17) previous history of cerebrovascular disease. In multiple regression analysis, multicollinearity was resolved by selecting covariates that reflects the status of the same disease; in the category of diabetes, covariates were (6) diabetes, (7) HbA_1c_ and (8) insulin use; in the category of hypertension, covariates were (9) hypertension and (10) ACEI or ARB use; in the category of dyslipidemia, covariates were (11) dyslipidemia, (12) LDL-C and (13) statin use; and in the category of heart failure, covariates were (14) congestive heart failure, (15) NYHA and (16) β-blocker use. From each category, the one covariate with the lowest *p* value and the highest odds ratio in the single regression analysis was selected. Finally, the absence of multi-collinearity among these 10 covariates was confirmed by variance inflation factors. Patient characteristics were stratified by quartiles of TMAO levels in serum and compared with a priori-defined 17 covariates, of which significant deviations were evaluated by linear regression analyses or Chi-square tests. First, the association between the number of infarcted coronary arteries and the quartiles of TMAO or each covariate was assessed with single ordered logistic regression models. Second, a multiple ordered logistic regression model was used to compute the association between the number of infarcted coronary arteries and quartiles of TMAO with adjustment by 10 covariates. Third, a multiple linear regression model instead of the multiple ordered logistic regression model was used to confirm the same trend with a different statistical model. To ensure robust main results in the sensitivity analyses, quintiles of TMAO levels, instead of quartiles of TMAO levels, and natural logarithm-transformed values of TMAO were used to confirm the main results. The risks were evaluated by odds ratios (ORs) and coefficients such as *β* with 95 % confidence intervals (95 % CIs). Values of *p* < 0.05 were considered significant. All data were statistically analyzed using STATA version 14.0 (STATA Corp., College Station, TX).

## Results

### Patient characteristics

Table 1 shows the baseline characteristics of the study population stratified by quartiles of serum TMAO levels. Median age was 68 years, and 30 % of patients were female. Older patients tended to have higher TMAO levels. However, smoking status and BMI did not differ among the quartiles of TMAO. Regarding CKD, advanced stages or maintenance hemodialysis were observed more frequently with higher quartiles of TMAO. Patients with diabetes and ACEI or ARB users were more common in quartiles 3 and 4 of TMAO compared with those in quartiles 1 and 2. An inverse association was identified between TMAO and LDL-C levels. No other significant covariates were found among the quartiles of TMAO. The characteristics of the 30 patients without TMAO data were not significantly different from those of the remaining 227 patients with TMAO data (data not shown).

**Table 1:** Patient characteristics stratified by quartiles of TMAO levels

*n*	Total	Quartile 1	Quartile 2	Quartile 3	Quartile 4	*p* value
	227	56	57	57	57	
Median	3.1	1.0	2.6	4.4	12.8	
25–75 %	1.7–6.0	0.6–1.3	2.0–2.8	3.5–4.9	8.6–17.2	
Age (year), median (25–75 %)	68 (61–74)	67 (60–73)	66 (58–74)	70 (62–74)	70 (64–76)	0.010*
Female, *n* (%)	69 (30)	17 (31)	19 (33)	20 (35)	13 (23)	0.50^a^
Smoking status (*n*)						0.48^a^
Never (%)	91 (43)	23 (48)	24 (43)	27 (49)	17 (32)	
Previously (%)	30 (14)	8 (17)	7 (13)	5 (9)	10 (19)	
Currently (%)	91 (43)	17 (35)	25 (45)	23 (42)	26 (49)	
BMI* (kg/m^2^), median (25–75 %)	23 (21–25)	22 (20–24)	23 (20–24)	23 (21–26)	23 (21–25)	0.31*
Chronic kidney disease stage, *n* (%)						<0.001^a^
Stage 1	11 (5)	5 (9)	3 (5)	1 (2)	2 (4)	
Stage 2	109 (48)	39 (70)	31 (54)	25 (44)	14 (25)	
Stage 3	77 (34)	12 (21)	19 (33)	27 (47)	19 (33)	
Stage 4	9 (4)	0 (0)	2 (4)	2 (4)	5 (9)	
Stage 5	3 (1)	0 (0)	0 (0)	1 (2)	2 (4)	
Stage 5D	18 (8)	0 (0)	2 (4)	1 (2)	15 (26)	
Diabetes, *n* (%)	62 (27)	10 (18)	10 (18)	20 (35)	22 (39)	0.014^a^
Hb_A1c_ (%), median (25–75 %)	5.5 (5.2–5.9)	5.4 (5.2–5.8)	5.3 (5.2–5.7)	5.5 (5.1–6.1)	5.5 (5.3–5.9)	0.16*
Insulin use, *n* (%)	9 (5)	1 (2)	0 (0)	3 (5)	5 (9)	0.08^a^
Hypertension, *n* (%)	177 (78)	41 (73)	39 (68)	48 (84)	49 (86)	0.07^a^
ACEI or ARB use, *n* (%)	134 (59)	32 (57)	23 (40)	40 (70)	39 (68)	0.004^a^
Dyslipidemia, *n* (%)	117 (52)	27 (48)	30 (53)	32 (56)	28 (49)	0.83^a^
LDL-C (mg/dL), median (25–75 %)	101 (80–126)	110 (91–130)	112 (94–136)	90 (78–113)	89 (69–111)	<0.001*
Statins use, *n* (%)	79 (39)	15 (33)	16 (30)	25 (48)	23 (43)	0.21^a^
Congestive heart failure, *n* (%)	42 (19)	12 (21)	8 (14)	12 (21)	10 (18)	0.72^a^
NYHA, *n* (%)						0.21^a^
0	1 (0.4)	0 (0)	1 (2)	0 (0)	0 (0)	
1	124 (55)	37 (66)	30 (53)	31 (54)	26 (46)	
2	83 (37)	16 (29)	23 (40)	22 (39)	22 (39)	
3	17 (7)	2 (4)	3 (5)	3 (5)	9 (16)	
4	2 (1)	1 (2)	0 (0)	1 (2)	0 (0)	
β-blocker use, *n* (%)	81 (36)	14 (25)	23 (40)	22 (39)	22 (39)	0.29^a^
Stroke						
History of cerebrovascular disease	31 (14)	6 (11)	6 (11)	8 (14)	11 (19)	0.49^a^

* *p* values were evaluated using a linear regression model

^a^Chi-square test

### Distribution of TMAO and its association with outcome

**Fig. 2:**
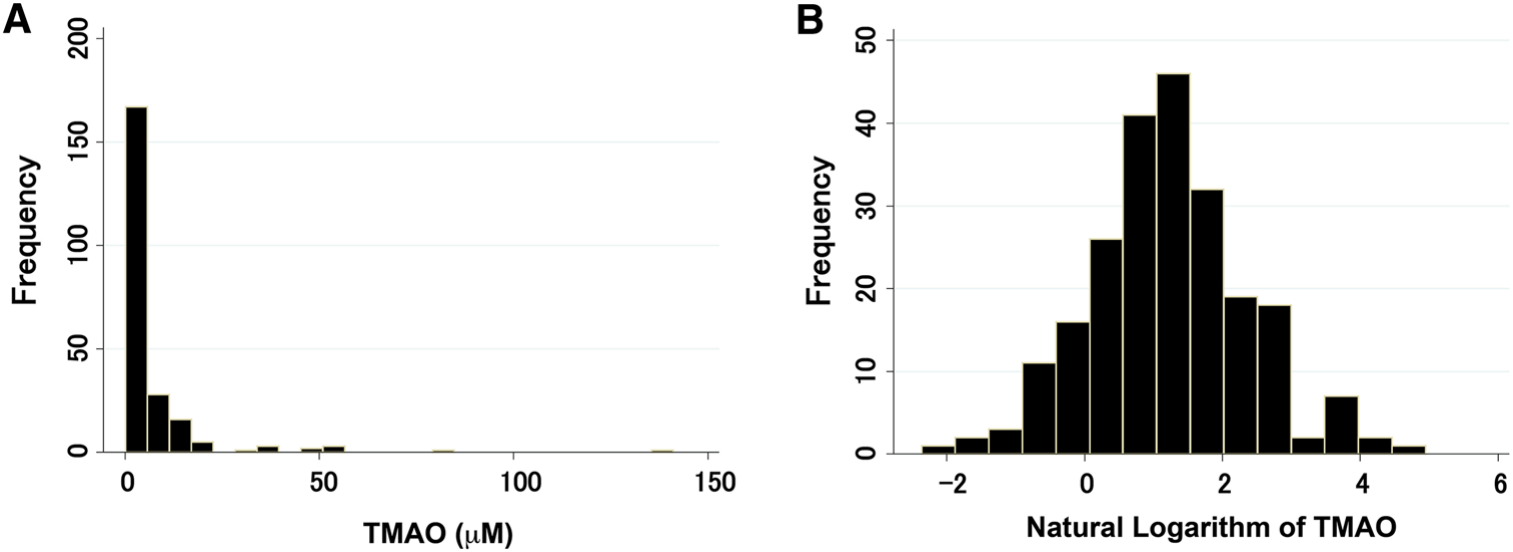
Distribution of serum TMAO levels. Histogram shows serum TMAO values (**a**) and natural logarithm-transformed values of TMAO (**b**) for all patients

Serum TMAO levels ranged from 0.09 to 141.2 μM (mean 6.96 μM, median 3.07 μM), with a non-normal distribution (Fig. 2a). However, the distribution of TMAO was normalized by natural logarithm transformation (Fig. 2b).

**Fig. 3:**
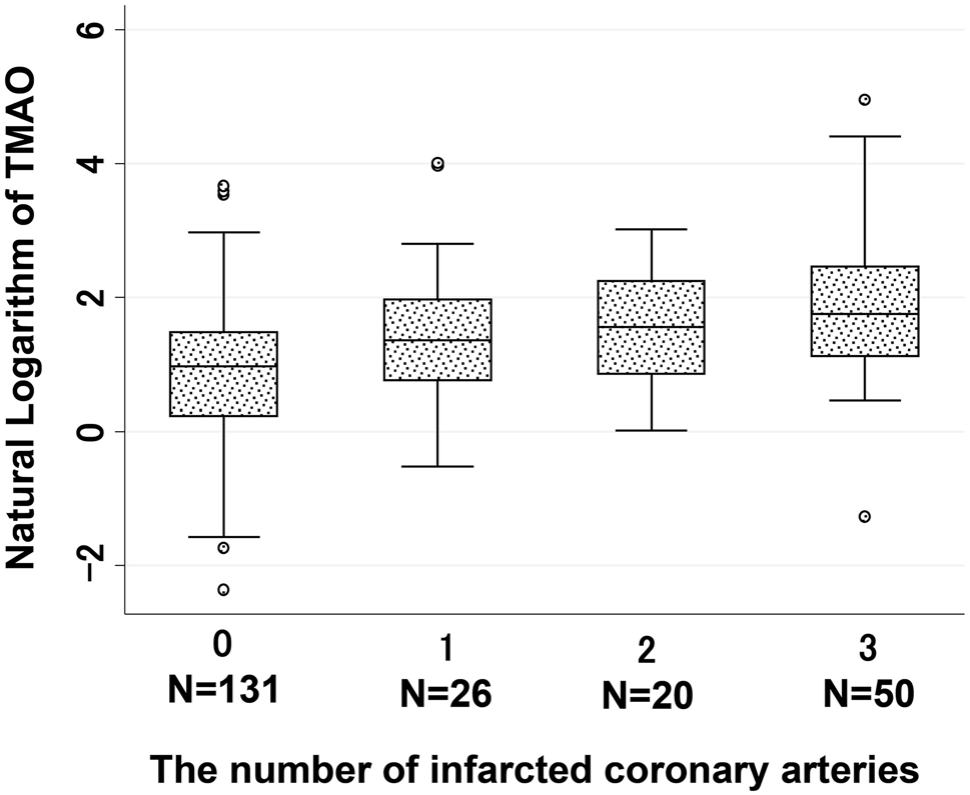
Association between serum TMAO levels and number of infarcted coronary arteries. *Box plots* show TMAO levels and the number of infarcted coronary arteries. TMAO levels are significantly associated with the number of infarcted coronary arteries. *Shaded areas of boxes* 25th and 75th percentiles; *thick line across each box* median; *whisker lines* 95 % confidence intervals for each category; *small circles* outliers

The outcome measure of the number of infarcted coronary arteries ranged from 0 to 3. The median (25–75 %) TMAO levels in patients who had zero, one, two, and three infarcted coronary arteries were 2.64 (1.26–4.42) μM, 3.94 (2.12–7.14) μM, 4.78 (2.35–9.43) μM, and 5.76 (3.05–11.7) μM, respectively. When the number of infarcted coronary arteries increased, the natural logarithm-transformed TMAO levels also increased significantly (Fig. 3) (simple linear regression analysis: *p* < 0.001).

### Association between TMAO levels and outcome measure

First, associations among the number of infarcted coronary arteries, quartiles of serum TMAO levels, and other covariates were analyzed (Table 2). In a single ordered logistic regression model, a significant and prominent increase in the number of infarcted coronary arteries was identified in the highest quartile of TMAO versus the lowest quartile (OR 8.66; 95 % CI 3.90–19.3; *p* < 0.001). The second and third quartiles were also higher than the lowest. In addition, male sex, higher BMI, diabetes, higher HbA1c, insulin use, hypertension, ACEI or ARB use, dyslipidemia, statin use, higher NYHA, β-blocker use, and a history of cerebrovascular disease were associated with an increased number of infarcted coronary arteries. Before multiple regression analysis, covariates were selected based on the results of single regression analysis. The selected covariates were: (1) age; (2) sex; (3) smoking status (never, previous, or current smoker); (4) BMI; (5) CKD stage; (6) insulin use; (7) hypertension; (8) dyslipidemia; (9) β-blocker use; and (10) previous history of cerebrovascular disease.

**Table 2:** Ordered logistic and linear regression models: association between quartiles of TMAO and number of infarcted coronary arteries

	Simple ordered logistic regression			Multiple ordered logistic regression			Multiple linear regression		
	OR	*p* value	95 % CI	OR	*p* value	95 % CI	*β*	*p* value	95 % CI
TMAO									
Quartile 1	Ref.			Ref.			Ref.		
Quartile 2	2.36	0.040	1.04–5.36	2.25	0.11	0.83–6.15	0.38	0.09	−0.06 to 0.81
Quartile 3	2.80	0.013	1.24–6.32	3.35	0.020	1.21–9.28	0.52	0.022	0.08 to 0.97
Quartile 4	8.66	<0.001	3.90–19.3	11.9	<0.001	3.88–36.7	1.33	<0.001	0.83 to 1.83
Age (years)	1.02	0.063	0.99–1.05	1.02	0.21	0.99–1.06	0.01	0.15	0 to 0.03
Female	0.47	0.007	0.28–0.82	0.41	0.046	0.17–0.98	−0.35	0.07	−0.73 to 0.03
Smoking status									
Never	Ref.			Ref.			Ref.		
Previous	1.92	0.08	0.92–4.04	0.80	0.70	0.27–2.40	−0.11	0.66	−0.61 to 0.38
Current	1.46	0.15	0.87–2.47	0.67	0.34	0.30–1.50	−0.20	0.30	−0.58 to 0.18
Body mass index (kg/m^2^)	1.14	<0.001	1.06–1.23	1.06	0.27	0.96–1.17	0.02	0.40	−0.03 to 0.07
Chronic kidney disease									
Stage 1	0.28	0.11	0.06–1.34	0.82	0.83	0.13–5.31	−0.05	0.91	−0.93 to 0.83
Stage 2	0.71	0.48	0.28–1.83	1.67	0.46	0.44–6.37	0.22	0.49	−0.42 to 0.87
Stage 3	0.79	0.63	0.30–2.08	0.83	0.79	0.22–3.17	−0.05	0.88	−0.69 to 0.59
Stage 4	0.87	0.84	0.20–3.67	0.54	0.51	0.09–3.37	−0.44	0.35	−1.37 to 0.49
Stage 5	1.00	1.00	0.13–7.59		1.00	0–	0		
Stage 5D	Ref.			Ref.			Ref		
Diabetes	5.81	<0.001	3.43–9.86						
HbA_1c_	2.82	<0.001	1.94–4.09						
Insulin use	17.5	<0.001	3.70–82.4	7.22	0.049	1.01–51.5	0.93	0.017	0.17 to 1.69
Hypertension	2.17	0.012	1.19–3.96	0.69	0.41	0.29–1.64	−0.19	0.34	−0.57 to 0.20
ACEI or ARB use	1.73	0.027	1.06–2.82						
Dyslipidemia	4.44	<0.001	2.66–7.41	3.58	<0.001	1.78–7.21	0.61	<0.001	0.28 to 0.93
LDL-C	1.00	0.34	0.99–1.00						
Statin use	3.90	<0.001	2.32–6.55						
Congestive heart failure	1.50	0.16	0.86–2.61						
NYHA	1.99	<0.001	1.38–2.85						
β-blocker use	3.39	<0.001	2.07–5.56	2.42	0.015	1.19–4.94	0.58	0.001	0.24 to 0.92
Cerebrovascular disease	2.70	0.006	1.33–5.45	1.81	0.25	0.66–4.93	0.27	0.28	−0.22 to 0.75

On multivariate adjustment using these 10 covariates, the association between quartiles of TMAO and the outcome measure was re-evaluated using a multiple ordered logistic regression model. A significantly increased number of infarcted coronary arteries was identified in the highest quartile of TMAO versus the lowest quartile (OR 11.9; 95 % CI 3.88–36.7; *p* < 0.001), independent of diabetes, dyslipidemia, and other covariates. Third, the association was reanalyzed with a multiple linear regression model, and similar results were obtained; a significantly increased number of infarcted coronary arteries was seen in the highest quartile of TMAO versus the lowest quartile (*β* 1.33; 95 % CI 0.83–1.83; *p* < 0.001), independent of other covariates.

Finally, we reconfirmed our findings using sensitivity analyses (Table 3). In a simple ordered logistic regression model, a higher quintile of TMAO was associated with an increased number of infarcted coronary arteries: the ORs of quintile 3, quintile 4, and quintile 5 (highest) compared with quintile 1 (lowest) were 2.63, 3.88, and 8.13, respectively. When the natural logarithm-transformed values of TMAO were used instead of the quintiles, the value of TMAO had a positive association with an increased number of infarcted coronary arteries. Even with multivariate adjustment using the 10 covariates selected, the trend was the same; namely, a significantly increased number of infarcted coronary arteries in the highest quintile of TMAO versus the lowest quintile (OR 14.1, 95 % CI 3.88–51.2, *p* < 0.001).

**Table 3:** Ordered logistic and linear regression models: association between quintiles of TMAO and number of infarcted coronary arteries

TMAO	Simple ordered logistic regression			Multiple ordered logistic regression^a^			Multiple linear regression^a^		
	OR	*p* value	95 % CI	OR	*p* value	95 % CI	*β*	*p* value	95 % CI
Quintile 1	Ref.			Ref.			Ref.		
Quintile 2	1.68	0.27	0.67–4.26	1.49	0.50	0.47–4.78	0.30	0.24	−0.20 to 0.79
Quintile 3	2.63	0.038	1.06–6.57	2.42	0.12	0.79–7.43	0.45	0.08	−0.05 to 0.96
Quintile 4	3.88	0.002	1.62–9.30	3.79	0.018	1.25–11.4	0.65	0.014	0.14 to 1.16
Quintile 5	8.13	<0.001	3.36–19.7	14.1	<0.001	3.88–51.2	1.42	<0.001	0.85 to 1.99
Natural logarithm of TMAO	2.03	<0.001	1.57–2.62	2.62	<0.001	1.73–3.96	0.46	<0.001	0.29 to 0.63

^a^Multivariate adjustment using 10 covariates: (1) age, (2) sex, (3) smoking status divided into three categories (never, previous, and current smoker), (4) BMI, (5) CKD stage, (6) insulin use, (7) hypertension, (8) dyslipidemia, (9) β-blocker use, and (10) previous history of cerebrovascular disease

## Discussion

In this study, higher serum levels of TMAO were strongly associated with an increased number of infarcted coronary arteries, even with adjustment for 10 covariates in patients who underwent cardiovascular surgery for CAD, VHD, or AoD. A significantly increased number of infarcted coronary arteries was seen in the highest quartile of TMAO as compared with the lowest quartile (OR 11.9, 95 % CI 3.88–36.7, *p* < 0.001), independent of previously known risk factors for CAD. These main results were considered robust, since both the highest quartile and the highest quintile (OR 14.1, 95 % CI 3.88–51.2, *p* < 0.001) showed associations with the outcome. In addition to previous studies by Tang et al. [2] and Koeth et al. [3], a recent cross-sectional study [9] and a cohort study [10] found that high TMAO serum levels increased the risks of cardiovascular events (death, myocardial infraction, stroke). In contrast to these articles, this study focused not on cardiovascular disease, but on the number of infarcted coronary arteries in patients who underwent cardiovascular surgery as a marker of atherosclerosis. In an animal model, supplementation with TMAO was demonstrated to be a direct inducer of atherosclerosis by augmenting macrophage cholesterol accumulation and foam cell formation [1], which may explain the mechanism behind why the number of infarcted coronary arteries increased in patients with higher levels of TMAO.

In this study, TMAO levels were elevated in patients with advanced CKD stages or treated with hemodialysis, which is consistent with previous articles [11–14]. TMAO clearance from serum is largely dependent on urinary excretion [15, 16]. On the other hand, in animal models, elevated dietary choline or TMAO led directly to progressive renal tubulo-interstitial fibrosis and dysfunction [11]. We, thus, hypothesized that impaired kidney function reduces renal excretion of TMAO, which increases serum TMAO levels. In turn, TMAO facilitates renal tubulo-interstitial fibrosis and dysfunction. These steps form a vicious circle and accelerate kidney dysfunction toward renal failure, which may cause further atherosclerosis and cardiovascular disease in CKD by raising TMAO levels. We also evaluated the synergistic effect between TMAO levels and CKD on CAD. However, no synergistic effect was observed in this study. These hypotheses thus need to be proven by conducting a long-term, prospective, cohort study involving a population without CKD.

In addition to the number of infarcted coronary arteries and CKD, the results of the present study implied associations between TMAO and other clinical factors. For example, TMAO levels were higher in patients with diabetes than in those without diabetes, which is also consistent with a recent article [17]. Dietary TMAO was shown to exacerbate impaired glucose tolerance, obstruct the hepatic insulin signaling pathway, and cause adipose tissue inflammation in mice fed a high-fat diet [18]. In addition, knockdown of FMO3, resulting in the absence of TMAO, can prevent the development of hyperglycemia and atherosclerosis in a diabetes mouse model [19], suggesting that the FMO3/TMAO pathway may play an important role in the pathophysiology of diabetes. In this study, no synergistic effect was observed between TMAO levels and DM on CAD. Regarding hypertension, there were no clear associations with TMAO. Similarly, a recent article reported that TMAO did not affect blood pressure in normotensive animals, but prolonged the hypertensive effect of angiotensin II [20]. Of interest, an inverse association was seen between LDL-C and TMAO levels. Dietary supplementation of TMAO or carnitine or choline in mice with intact intestinal microbiota significantly reduced reverse cholesterol transport in vivo [3]. Recently, the TMAO-generating enzyme FMO3 was found to be a central regulator of cholesterol balance [21]. Although high TMAO levels were reportedly observed in patients with heart failure [22, 23] or associated with its severity [24], no significant differences were seen in this study.

To the best of our knowledge, this is the first report to demonstrate the association between TMAO and kidney function and atherosclerosis in a Japanese population. However, this study has several limitations. (1) Because this study had a cross-sectional design, causal relationships among impaired renal function, high TMAO levels, and the number of infarcted coronary arteries can only be hypothesized, but need to be confirmed. (2) No healthy control group was included in this study, so the results may be applicable to patients who undergo cardiovascular surgery for CAD, VHD, or AoD, rather than the general population. (3) The amount of serum obtained from 30 of the patients was insufficient to measure TMAO levels, which may have caused selection bias, although the background characteristics of these 30 patients did not differ significantly from those of the 227 patients for whom data were analyzed.

Gut microbiota have recently become an exciting new topic due to the important roles played in digestion [25], metabolism [26], and immune function [27, 28], and a widespread impact beyond the gastrointestinal tract [29]. Associations between gut microbiota and renal disease have not been studied in detail from the viewpoint of renal science. However, the microbial flora is altered in patients with CKD compared with healthy individuals [30–32]. The gut microbiome should, therefore, be thoroughly investigated to increase our understanding of the pathogenesis of atherosclerosis and cardiovascular disease.

## Conclusions

Higher serum TMAO levels may be associated with an increased number of infarcted coronary arteries in patients who undergo cardiovascular surgery
